# Clinical Formulation Bridging of Gefapixant, a P2X3‐Receptor Antagonist, for the Treatment of Chronic Cough

**DOI:** 10.1002/cpdd.1105

**Published:** 2022-05-05

**Authors:** Pranav Gupta, Azher Hussain, Anthony P. Ford, Steven Smith, Jesse C. Nussbaum, Aubrey Stoch, Marian Iwamoto

**Affiliations:** ^1^ Merck & Co., Inc. Kenilworth New Jersey USA; ^2^ CuraSen Therapeutics San Carols California USA; ^3^ Afferent Pharmaceuticals, Inc. San Mateo California USA

**Keywords:** bioavailability, bioequivalence, chronic cough, formulation bridging, gefapixant

## Abstract

Gefapixant is a P2X3‐receptor antagonist being developed for treatment of refractory or unexplained chronic cough. Four phase 1 studies were conducted in healthy participants that bridged the early‐phase gefapixant formulation (F01) to the phase 3 (F04A) and intended commercial (F04B) formulations. In addition, food and proton pump inhibitor (PPI) coadministration effects on gefapixant exposure were assessed. The gefapixant free base formulation (F01) was used in the initial early‐phase clinical studies. Adding citric acid to the F01 formulation (to generate F02) enhanced drug solubilization, resulting in similar bioavailability and mitigating food and gastric pH effects. The subsequently developed gefapixant citrate salt formulation (F04) achieved exposures that were comparable to F02 in the fed state (90%CIs of geometric mean ratios for area under the plasma concentration–time curve from time 0 extrapolated to infinity and maximum observed concentration were within 0.80 and 1.25) and were not meaningfully affected by food or PPIs (90%CIs of geometric mean ratios for area under the plasma concentration–time curve from time 0 extrapolated to infinity and maximum observed concentration were within 0.80 and 1.25). Minor compositional changes were made to generate the F04A and F04B formulations. In vitro dissolution studies were used to bridge F04 to F04A, and clinical bioequivalence was then established between F04A and F04B. These data support use of the proposed commercial gefapixant formulation without significant food and PPI effects.

Chronic cough is defined as cough lasting >8 weeks according to published guidelines from the American College of Chest Physicians and the European Respiratory Society.^1^, ^2^ A subset of patients with chronic cough have a cough that persists despite treatment of presumed underlying conditions associated with cough (refractory chronic cough [RCC]) or a cough for which no underlying cause can be identified (unexplained chronic cough [UCC]); there are no effective and safe treatments with indications for RCC or UCC, reflecting a major unmet need.

Previous research has demonstrated that the cough reflex is partially initiated by the C‐fibers of the vagus nerve, and preclinical studies suggest that inhibiting the P2X3 receptor along the C‐fibers blocks the cough response without interfering with the protective function of cough.^3^, ^4^, ^5^ Thus, the P2X3 receptor has emerged as a potential therapeutic target for treatment of chronic cough. Gefapixant is a nonnarcotic, small‐molecule inhibitor of the P2X3 receptor being developed for treatment of RCC or UCC.^6^, ^7^, ^8^, ^9^, ^10^ Clinical efficacy and safety of gefapixant was demonstrated in 2 recent phase 3 trials (COUGH‐1 and COUGH‐2; ClinicalTrials.gov identifiers, NCT03449134 and NCT03449147, respectively), in which gefapixant significantly reduced 24‐hour cough frequency and improved cough‐related quality of life in patients with RCC or UCC.^11^

The disposition of gefapixant has been described separately; characterization of the pharmacokinetics of gefapixant demonstrated that the drug is rapidly absorbed, with time to maximum observed plasma concentration (t_max_) ranging from 1 to 4 hours and a terminal half‐life (t_1/2_) of ≈7 hours.^12^ Elimination of gefapixant primarily occurs via renal excretion of the intact drug, which is at least partially mediated by multidrug and toxin extrusion protein 1 (MATE1) and MATE2K.^12^, ^13^ Excreted metabolites account for only 14% of the gefapixant dose, with identified metabolites suggesting that gefapixant undergoes oxidation and direct glucuronidation.^12^ As gefapixant clearance via metabolism is minor, and inhibiting MATE1/2K via pyrimethamine does not meaningfully increase gefapixant exposure, the potential for clinically meaningful drug‐drug interactions between gefapixant and inhibitors or inducers of cytochrome P450, uridine 5′‐diphosphoglucuronic acid glucuronosyltransferase, or MATE1/2K is considered to be low.^12^, ^13^ Similarly, there was no effect of twice‐daily gefapixant 45 mg on the pharmacokinetics of pitavastatin, a clinical probe substrate of organic anion transporter P1B1 activity, suggesting that clinically meaningful drug‐drug interactions between gefapixant and organic anion transporter P1B1 inhibitors are unlikely.^14^

The formulation of gefapixant has evolved over the course of development. Five formulations with 2 different active pharmaceutical ingredient (API) forms of gefapixant were used during clinical development and/or for commercial use: F01, F02, F04, F04A, and F04B. The original gefapixant formulation, F01, was developed as a free base formulation in early‐phase clinical trials. The F02 gefapixant formulation was also a free base formulation, with the addition of anhydrous citric acid as an acidulant; this formulation was developed to address gastric pH and food effects. Citric acid was used as an acidulant to enhance drug bioavailability through increasing drug solubility by lowering the stomach microenvironmental pH around the dissolving dosage form^15^ and by formation of an in situ citrate salt that has significantly greater solubility than the corresponding free base. The F02 formulation was used in the phase 2 trials for gefapixant in RCC and UCC.^9^, ^10^ A new API, gefapixant citrate salt, was used in F04 formulations to enhance drug solubilization compared with the free base formulation F01 and to achieve rapid and complete dissolution of gefapixant independent of pH conditions. Additionally, the use of gefapixant citrate salt prevented the presence of different API phases (free base and citrate salt forms) observed in the F02 formulation, thereby resulting in a stable and robust drug product with predictable bioperformance. The same F04 API (gefapixant citrate salt) was used in F04A and F04B, with minor changes in certain excipients and changes in the film coating composition. The F04A formulation (containing citric acid as an acidulant and the disintegrant crospovidone type A) was developed to improve processability and was used in 2 phase 3 clinical trials. The F04B formulation (without citric acid and containing the disintegrant crospovidone type B) was developed for commercial use to resolve tablet elegance issues observed during stability evaluation with the F04A formulation.

A series of relative bioavailability studies was performed to bridge formulations of gefapixant from the original fit‐for‐purpose formulations to the final proposed commercial formulation. Herein, we describe 4 phase 1 clinical studies using a formulation‐bridging strategy originating with the gefapixant free base formulation and resulting in the commercial gefapixant citrate salt–based formulation. Food and gastric pH effects (via administering the proton pump inhibitor [PPI] omeprazole) were also assessed. The overall strategy for bridging gefapixant formulations is shown in Figure 1.

**Figure 1:**
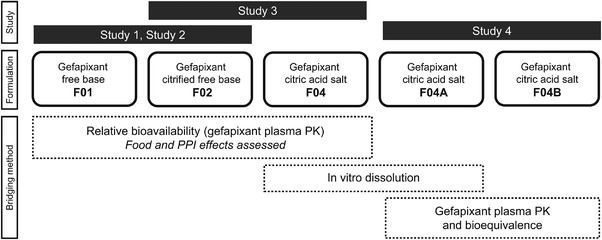
Bridging strategy for gefapixant formulations. Effects of food and a PPI were assessed and compared between formulations F01, F02, and F04 in addition to relative bioavailability. As no changes to the API occurred once formulation F04 had been generated, gefapixant formulations F04 and F04A were bridged using in vitro dissolution studies (data not included). Bioequivalence was examined for F04A and F04B. API, active pharmaceutical ingredient; PK, pharmacokinetic; PPI, proton pump inhibitor.

## Methods

### Study Objectives and Design

Four single‐center, open‐label studies were conducted in healthy adult participants. The key objectives, pharmacokinetic (PK) end points, and PK sampling time points of the 4 studies are shown in Table 1. All studies were conducted in compliance with the ethical principles set forth in the Declaration of Helsinki and according to the guidelines resulting from the International Conference on Harmonisation and Good Clinical Practice. Written informed consent was obtained from all study participants. Protocols for study 1 and study 2 were reviewed and approved by the institutional review board (IRB) Chesapeake Research Review, Inc (now Advarra Inc, Columbia, Maryland). The protocol for study 3 was reviewed and approved by the Salus IRB (Austin, Texas). The protocol for study 4 was reviewed and approved by the IRB Advarra Inc (Columbia, Maryland).

**Table 1:** Clinical Gefapixant Formulation‐Bridging Studies

Study ID/Location	Formulations	Key Study Objectives	Key Primary PK End Points	PK Sampling Time Points
Study 1 (NCT02229877) Celerion (Tempe, Arizona)	F01, F02	Compare bioavailability, in presence and absence of omeprazole, of F01 50 and 150 mg after multiple doses in fed and fasted states Compare bioavailability, in presence and absence of omeprazole, of F02 150 mg after multiple doses in fed and fasted states	Plasma PK parameters for gefapixant (AUC_0‐12_ and C_max_)	Before dosing (time 0) and 1, 2, 3, 5, 8, and 12 h after dosing
Study 2 (NCT02492776) Celerion (Tempe, Arizona)	F01, F02	Compare bioavailability of F01 50 mg and F02 25 and 50 mg after multiple doses in the fed state Compare bioavailability, in presence and absence of omeprazole, of F01 50 mg and F02 25 and 50 mg after multiple doses in the fed state	Plasma PK parameters for gefapixant (AUC_0‐12_ and C_max_)	Before dosing (time 0) and 1, 2, 3, 5, 8, and 12 h after dosing
Study 3^a^ Pharma Medica Research Inc (St Charles, Missouri)	F02, F04	Compare bioavailability of F02 and F04 50 mg (single dose) in fed and fasted states Compare bioavailability, in presence and absence of omeprazole, of F04 50 mg (single dose) in the fasted state	Plasma PK parameters for gefapixant (AUC_0‐12_, AUC_0‐∞_, and C_max_)	Before dosing (time 0) and 0.25, 0.5, 1, 2, 3, 4, 6, 8, 12, 16, 24, 36, and 48 h after dosing
Study 4^a^ Celerion (Tempe, Arizona)	F04A, F04B	Compare bioavailability of F04A and F04B 45 mg (single dose) in the fasted state Compare bioavailability of F04A and F04B 15 mg (single dose) in the fasted state	Plasma PK parameters for gefapixant (AUC_0‐last_, AUC_0‐∞_, C_max_)	Before dosing (time 0) and 0.25, 0.5, 1, 1.5, 2, 3, 4, 6, 8, 12, 16, 24, 32, 48, and 72 h after dosing

AUC_0‐∞_, area under the plasma concentration–time curve from time 0 extrapolated to infinity; AUC_0‐12_, area under the plasma concentration–time curve from time 0 to 12 h; AUC_0‐last_, area under the plasma concentration–time curve from time 0 to the time of the last quantifiable sample; C_max_, maximum observed gefapixant concentration; PK, pharmacokinetic.

a Studies 3 and 4 were not required to be formally registered.

#### Study 1

Study 1 consisted of 2 parts (part A and part B). Eligible participants were administered the following treatments: (1) placebo every 12 hours taken after a moderate‐fat meal and administered for 1 day; (2) gefapixant F01 25 mg every 12 hours taken after a moderate‐fat meal and administered for 2 days; (3) gefapixant F01 50 mg every 12 hours taken after a moderate‐fat meal and administered for 2 days; (4) gefapixant F01 50 mg every 12 hours taken in the fasted state and administered for 2 days; (5) gefapixant F01 150 mg every 12 hours taken after a moderate‐fat meal and administered for 2 days; and (6) gefapixant F01 150 mg every 12 hours taken in the fasted state and administered for 2 days. Omeprazole 40 mg was administered 2 hours before the following treatments: (7) gefapixant F01 50 mg every 12 hours taken after a moderate‐fat meal and administered for 2 days; (8) gefapixant F01 50 mg every 12 hours taken in the fasted state and administered for 2 days; (9) gefapixant F01 150 mg every 12 hours taken after a moderate‐fat meal and administered for 2 days; (10) gefapixant F01 150 mg every 12 hours taken in the fasted state and administered for 2 days; and (11) 1 dose of placebo taken after a moderate‐fat meal. There was no washout period between treatments.

In part B, a subset of participants who previously participated in part A received the following treatments: (1) gefapixant F02 150 mg every 12 hours taken after a moderate‐fat meal for 2 doses; (2) gefapixant F02 150 mg every 12 hours taken in the fasted state for 2 doses; (3) gefapixant F02 150 mg every 12 hours taken after a moderate‐fat meal and 2 hours after administration of omeprazole 40 mg for at least 2 days; and (4) gefapixant F02 150 mg every 12 hours taken in the fasted state and 2 hours after administration of omeprazole 40 mg for at least 2 days. There was no washout period between treatments.

For both part A and part B, a meal was administered 30 minutes before dosing in fed states. Fasted states were defined as a period of overnight fasting, with no food permitted for at least 2 hours after morning dosing (and at least 4 hours on days of plasma collection). Evening dosing occurred after fasting for at least 2 hours; no food was permitted for at least 2 hours after dosing. A high dose of omeprazole (40 mg every 12 hours) was used to test the difference in F02 bioperformance in the presence of a PPI; a more common dose of omeprazole (40 mg once daily or 20 mg once daily) was used in subsequent studies.

#### Study 2

In study 1, part B, only the 150‐mg dose level of F02 was assessed. To further enhance the data set, study 2 was conducted to compare additional dose levels of F01 and F02. In study 2, healthy participants received the following: (1) gefapixant F02 25 mg every 12 hours for 1 day; (2) gefapixant F02 50 mg every 12 hours for 2 days; (3) gefapixant F01 50 mg every 12 hours for 2 days; (4) gefapixant F02 25 mg every 12 hours with omeprazole 20 mg once daily for 4 days; (5) gefapixant F01 50 mg every 12 hours with omeprazole 20 mg once daily for 2 days; and (6) gefapixant F02 50 mg every 12 hours with omeprazole 20 mg once daily for 2 days. Omeprazole was administered in the mornings on days 7 to 14, 2 hours before the morning gefapixant dose. Participants received gefapixant in the fed state in all treatment periods; meals were administered 30 minutes before dosing.

#### Study 3

In Study 3, healthy participants received the following drug treatments in 5 periods: (1) gefapixant F02 50 mg administered in the fasted state; (2) gefapixant F04 50 mg administered in the fasted state; (3) gefapixant F04 50 mg administered in the fed state; (4) omeprazole 40 mg administered once daily for 5 days in the fasted state with gefapixant F04 50 mg administered 2 hours after the last omeprazole dose on the fifth day; and (5) gefapixant F02 50 mg administered in the fed state. For treatments 1 and 2 in the fasted state, participants fasted for at least 10 hours before drug administration, and no food was allowed for at least 4 hours after dosing. For treatments 3 and 5 in the fed state, participants fasted overnight for at least 10 hours and consumed a high‐fat, high‐calorie breakfast 30 minutes before drug administration. For treatment 4, participants fasted for at least 8 hours before omeprazole administration and until at least 2 hours after dosing (except for the fifth day, during which food was restricted until at least 4 hours after administration of gefapixant F04 50 mg). There was a 7‐day washout period between each drug administration in the first 4 treatments and a 22‐day washout period between treatments 4 and 5.

#### Study 4

Study 4 consisted of 2 parts. In part 1, healthy participants received gefapixant F04A 45 mg or gefapixant F04B 45 mg in a randomized, crossover manner. In part 2, healthy participants received gefapixant F04A 15 mg or gefapixant F04B 15 mg in a randomized, crossover manner. These doses were selected for the phase 3 gefapixant development program on the basis of quantitative modeling of dose‐response estimates based on efficacy, safety, and tolerability end points from previous phase 2 studies.^16^ In both parts of study 4, a 5‐day washout period separated the 2 treatments. Participants fasted from all food and drinks, except water, for at least 10 hours before and at least 4 hours after dosing.

### Participants

All 4 studies included healthy adult participants aged 18 to 55 years (studies 1 and 2) or 65 years (studies 3 and 4) who had a body mass index of >18.5 and <32.0 kg/m^2^ (studies 1 and 2), ≥19.0 and ≤33.0 kg/m^2^ (study 3), or ≥18.0 and ≤32.0 kg/m^2^ (study 4). Individuals with illness that would have affected drug absorption, metabolism, or excretion were excluded. Demographics for participants in all 4 studies are included in the Table S1.

### Assessments

#### Bioanalysis

The time points used for blood draws for PK analyses for each study can be found in Table 1. Plasma samples were analyzed by inVentiv Health Clinique (studies 1, 2, and 3; Montréal, Canada) and Syneos Health Clinique (formerly inVentiv Health Clinique; study 4; Québec City, Canada). The bioanalytic methods used in all 4 studies were previously reported.^13^ The lower limit of quantitation for this method was 10 ng/mL, with a linear calibration range from 10 to 10 000 ng/mL for studies 1 to 3 and 10 to 1000 ng/mL for study 4.

#### Pharmacokinetic Analysis

PK parameters of interest included area under the plasma concentration–time curve (AUC) from time 0 to 12 hours (AUC_0‐12_), AUC from time 0 to the time of the last quantifiable sample (AUC_0‐last_), AUC from time 0 extrapolated to infinity (AUC_0‐∞_), maximum observed gefapixant concentration (C_max_), t_max_, and t_1/2_, as appropriate. PK parameter values were calculated using standard noncompartmental methods from gefapixant plasma concentration–time data using Phoenix WinNonlin (Certara, Princeton, New Jersey), version 6.3 (studies 1 and 2), 6.4 (study 3), or 7.0 (study 4). AUC_0‐∞_ and AUC_0‐12_ were calculated using the linear trapezoidal method for ascending concentrations and the log trapezoidal method for descending concentrations (*linear up, log down* calculation methods).

C_max_ and t_max_ were derived directly from bioanalytical data. At least 3 consecutive time points in the terminal phase, excluding t_max_, were used for the apparent t_1/2_ determination.

### Safety Analysis

Safety and tolerability for all studies were determined using clinical assessments, including physical examinations, vital signs, standard laboratory tests, 12‐lead electrocardiograms, and adverse events (AEs).

### Statistical Analysis

SAS (SAS Institute, Cary, North Carolina) version 9.3.4 (study 1), 9.3 (study 2), or 9.4 (study 4) was used for statistical analyses. In studies 1 and 2, analysis of variance was performed on individual natural log‐transformed AUC and C_max_ values to compare formulation bioavailability and determine any effects of food or omeprazole on pharmacokinetics. The analysis of variance model included treatment and sequence as fixed effects and subject within sequence as a random effect. Geometric mean ratios (GMRs) and 90%CIs were calculated for AUC and C_max_. In study 3, natural log‐transformed individual PK values were evaluated using a linear mixed‐effects model with treatment as fixed effect. In study 4, natural log‐transformed individual PK values were evaluated using a linear mixed‐effects model with treatment and period as fixed effects. Values below the limit of quantitation were treated as 0 in calculating the arithmetic mean. The least‐square means and CIs were exponentiated to obtain CIs for the true GMR AUC and C_max_. In study 4, bioequivalence was confirmed if the 90%CIs of the GMRs for PK parameters (AUC_0‐∞_, AUC_0‐last_, and C_max_) fell within prespecified bounds (0.80‐1.25).

## Results

### Formulation Bridging Gefapixant F01 to F02

#### Study 1

**Figure 2:**
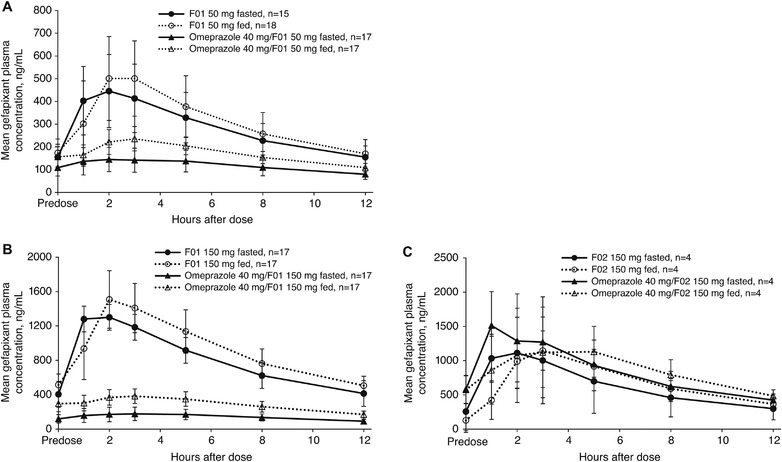
Mean (standard deviation) gefapixant plasma concentrations over time in healthy adult participants following administration of gefapixant (A) F01 50 mg, (B) F01 150 mg, or (C) F02 150 mg with or without food and with or without coadministration of omeprazole 40 mg every 12 hours for 2 days.

A total of 18 participants were enrolled in study 1, and all were included in the safety analysis; 1 participant discontinued because of mild ageusia and mild dizziness. Mean gefapixant plasma AUC_0‐12_ and C_max_ values are presented in Table 2; mean gefapixant plasma concentration vs time curves are shown in Figure 2.

**Table 2:** Summary of Statistical Comparisons of Gefapixant Pharmacokinetics for Formulations F01 and F02 After Administering Gefapixant With or Without Omeprazole to Healthy Adult Participants (Assessed in Fed and Fasted States)

Parameter	n	Arithmetic Mean (SD)	Geometric LSM (Test)	n	Arithmetic Mean (SD)	Geometric LSM (Reference)	GMR (Test/Reference) (90%CI)	Intrasubject CV, %^a^
**F01 formulation: intragastric pH effect**								
	**F01 50 mg every 12 h in fed state** **+ omeprazole 40 mg** **^b^**			**F01 50 mg every 12 h in fed state**				
AUC_0‐12_, ng · h/mL	17	2074 (391)	1814	18	3811 (1113)	3472	0.52 (0.46‐0.60)	24.2
C_max_, ng/mL	17	242 (48)	212	18	530 (163)	480	0.44 (0.38‐0.51)	27.2
	**F01 150 mg every 12 h in fed state +** **omeprazole 40 mg** **^b^**			**F01 150 mg every 12 h in fed state**				
AUC_0‐12_, ng · h/mL	17	3513 (801)	3052	17	11 270 (2142)	9862	0.31 (0.27‐0.35)	24.2
C_max_, ng/mL	17	395 (85)	345	17	1554 (301)	1361	0.25 (0.22‐0.30)	27.2
	**F01 50 mg every 12 h in fasted state** **+ omeprazole 40 mg** **^b^**			**F01 50 mg every 12 h in fasted state**				
AUC_0‐12_, ng · h/mL	17	1433 (487)	1217	15^c^	3461 (1366)	2994	0.41 (0.35‐0.47)	24.2
C_max_, ng/mL	17	156 (57)	131	15^c^	470 (193)	402	0.33 (0.28‐0.38)	27.2
	**F01 150 mg every 12 h in fasted state** **+ omeprazole 40 mg** **^b^**			**F01 150 mg every 12 h in fasted state**				
AUC_0‐12_, ng · h/mL	17	1732 (595)	1470	17	9818 (3720)	8038	0.18 (0.16‐0.21)	24.2
C_max_, ng/mL	17	191 (81)	160	17	1434 (568)	1161	0.14 (0.12‐0.16)	27.2

**F02 formulation: intragastric pH effect**								
	**F02 150 mg every 12 h in fed state** **+ omeprazole 40 mg** **^b^**			**F02 150 mg every 12 h in fed state**				
AUC_0‐12_, ng · h/mL	4	10 422 (3747)	9984	4	8244 (831)	8213	1.22 (0.91‐1.62)	22.4
C_max_, ng/mL	4	1336 (496)	1273	4	1256 (263)	1236	1.03 (0.85‐1.24)	14.6
	**F02 150 mg every 12 h in fasted state** **+ omeprazole 40 mg** **^b^**			**F02 150 mg every 12 h in fasted state**				
AUC_0‐12_, ng · h/mL	4	10 311 (1921)	10167	4	7704 (4386)	6974	1.46 (1.09‐1.94)	22.4
C_max_, ng/mL	4	1666 (367)	1633	4	1169 (527)	1096	1.49 (1.23‐1.80)	14.6

AUC_0‐12_, area under the plasma concentration–time curve from time 0 to 12 h; C_max_, maximum observed gefapixant concentration; CV, coefficient of variance; GMR, geometric mean ratio; LSM, least‐square mean; SD, standard deviation.

a The intrasubject CV percentage = 100√(e^[residual variance] − 1^).

b Omeprazole 40 mg coadministered with gefapixant for 2 days.

c Three participants were not included in the summary statistics for this treatment because of a major protocol deviation.

In participants who received either gefapixant F01 50 or 150 mg Q12h for 2 days in the presence of omeprazole 40 mg every 12 hours, gefapixant AUC_0‐12_ and C_max_ were significantly lower compared with F01 in the absence of omeprazole in both fed and fasted states; administration of F01 in the presence of omeprazole resulted in decreases in gefapixant AUC_0‐12_ and C_max_ by ≈2‐ to 5‐fold and 2‐ to 7‐fold, respectively, with larger fold decreases occurring at the 150‐mg dose and in the fasted state. In 4 participants receiving gefapixant F02 150 mg every 12 hours, coadministration of omeprazole 40 mg every 12 hours slightly increased gefapixant AUC_0‐12_ and had no meaningful effect on C_max_ in the fed state compared with F02 150 mg in the absence of omeprazole (Table 2; Figure 2). In the fasted state, coadministration of omeprazole 40 mg every 12 hours with F02 150 mg increased both the AUC_0‐12_ and C_max_ of gefapixant compared with F02 150 mg alone (Table 2). The overall changes in gefapixant AUC_0‐12_ and C_max_ after F02 150 mg administration in the presence of omeprazole were no more than 1.5‐fold.

Gefapixant AUC_0‐12_ and C_max_ were generally higher with gefapixant F01 50 or 150 mg every 12 hours under fed compared with fasted states, with a ≈20% increase in exposure (Table 3). After omeprazole administration, the effect was more pronounced, with a ≈2‐fold increase in exposure in the fed state. Similarly, in the 4 participants receiving F02 150 mg every 12 hours administered in the fed state, gefapixant AUC_0‐12_ and C_max_ were slightly higher than in the fasted state, whereas the opposite trend was observed when administered with omeprazole (Table 3). However, all changes in gefapixant exposure after F02 administration in the fed vs fasted states were no more than a ≈25% reduction.

**Table 3:** Summary of Statistical Comparisons of Gefapixant Pharmacokinetics for Formulations F01 and F02 After Administering Gefapixant to Healthy Adult Participants (Assessed in Fed and Fasted States)

Parameter	n	Geometric LSM	n	Geometric LSM	GMR (Fed/Fasted) (90%CI)	Intrasubject CV, %^a^
**F01 formulation: food effects**						
	**F01 50 mg every 12 h in fed state**		**F01 50 mg every 12 h in fasted state**			
AUC_0‐12_, ng · h/mL	18	3472	15^b^	2994	1.16 (1.01‐1.33)	24.2
C_max_, ng/mL	18	480	15^b^	402	1.19 (1.02‐1.39)	27.2
	**F01 150 mg every 12 h in fed state**		**F01 150 mg every 12 h in fasted state**			
AUC_0‐12_, ng · h/mL	17	9862	17	8038	1.23 (1.07‐1.41)	24.2
C_max_, ng/mL	17	1361	17	1161	1.17 (1.01‐1.36)	27.2
	**F01 50 mg every 12 h + omeprazole 40 mg every 12 h in fed state** **^c^**		**F01 50 mg every 12 h + omeprazole 40 mg every 12 h in fasted state** **^c^**			
AUC_0‐12_, ng · h/mL	17	1814	17	1217	1.49 (1.30‐1.71)	24.2
C_max_, ng/mL	17	212	17	131	1.61 (1.38‐1.88)	27.2
	**F01 150 mg every 12 h + omeprazole 40 mg every 12 h in fed state** **^c^**		**F01 150 mg every 12 h + omeprazole 40 mg every 12 h in fasted state** **^c^**			
AUC_0‐12_, ng · h/mL	17	3052	17	1470	2.08 (1.81‐2.38)	24.2
C_max_, ng/mL	17	345	17	160	2.16 (1.86‐2.52)	27.2

**F02 formulation: food effects**						
	**F02 150 mg every 12 h in fed state**		**F02 150 mg every 12 h in fasted state**			
AUC_0‐12_, ng · h/mL	4	8213	4	6974	1.18 (0.88‐1.57)	22.4
C_max_, ng/mL	4	1236	4	1096	1.13 (0.93‐1.36)	14.6
	**F02 150 mg every 12 h + omeprazole 40 mg every 12 h in fed state** **^d^**		**F02 150 mg every 12 h + omeprazole 40 mg every 12 h in fasted state** **^d^**			
AUC_0‐12_, ng · h/mL	4	9984	4	10 167	0.98 (0.74‐1.31)	22.4
C_max_, ng/mL	4	1274	4	1633	0.78 (0.65‐0.94)	14.6

AUC_0‐12_, area under the plasma concentration–time curve from time 0 to 12 h; C_max_, maximum observed gefapixant concentration; CV, coefficient of variance; GMR, geometric mean ratio; LSM, least‐square mean.

a The intrasubject CV percentage = 100√(e^[residual variance] − 1^).

b Three participants were not included in the summary statistics for this treatment because of a major protocol deviation.

c Coadministered for 2 days.

d F02 150 mg every 12 h taken after administration of omeprazole 40 mg every 12 h for 2 days.

#### Study 2

**Figure 3:**
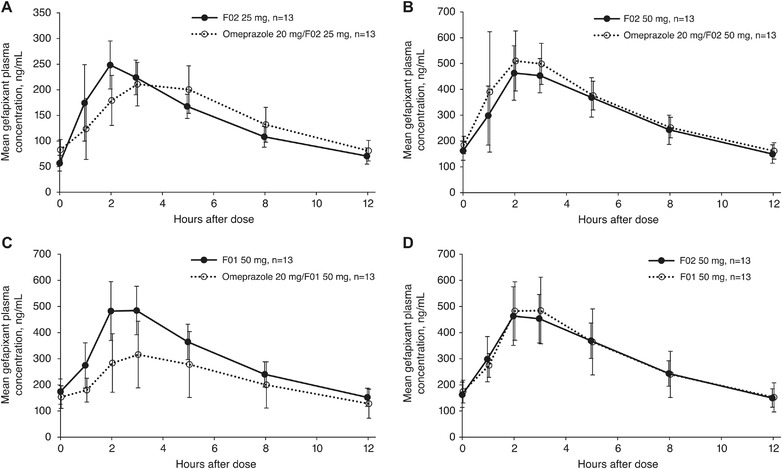
Mean (standard deviation) gefapixant plasma concentrations over time in healthy adult participants following administration of gefapixant (A) F02 25 mg every 12 hours for 1 day alone or with omeprazole 20 mg once daily for 4 days, (B) F02 50 mg every 12 hours alone or with omeprazole 20 mg once daily for 2 days, (C) F01 50 mg every 12 hours alone or with omeprazole 20 mg once daily for 2 days, and (D) F01 50 mg and F02 50 mg every 12 hours for 2 days. In all treatments, gefapixant was administered in the fed state.

A total of 14 participants were enrolled in the study, and all received at least 1 dose of study drug and were included in the safety analysis; 1 participant withdrew after only 1 dose of F02 25 mg for personal reasons. Mean gefapixant plasma concentration vs time curves are presented in Figure 3; AUC_0‐12_ and C_max_ values are presented in Table 4.

**Table 4:** Summary of Statistical Comparisons of Gefapixant Pharmacokinetics for Formulations F01 and F02 After Administering Gefapixant With or Without Omeprazole to Healthy Adult Participants (Assessed in the Fed State)

	With PPI^a^			Without PPI				
Parameter	n	Arithmetic Mean (SD)	Geometric LSM	n	Arithmetic Mean (SD)	Geometric LSM	GMR (With PPI/Without PPI) (90%CI)	Intrasubject CV, %^b^
**F02 25 mg every 12 h in fed state**								
AUC_0‐12_, ng · h/mL	13	1776 (277)	1766	13	1719 (276)	1708	1.03 (0.94‐1.13)	14.1
C_max_, ng/mL	13	231 (40)	229	13	257 (43)	256	0.90 (0.82‐0.99)	14.5
**F02 50 mg every 12 h in fed state**								
AUC_0‐12_, ng · h/mL	13	3880 (571)	3863	13	3580 (498)	3569	1.08 (0.99‐1.19)	14.1
C_max_, ng/mL	13	577 (125)	569	13	510 (51)	511	1.11 (1.01‐1.22)	14.5
**F01 50 mg every 12 h in fed state**								
AUC_0‐12_, ng · h/mL	13	2661 (1042)	2487	13	3612 (555)	3594	0.69 (0.63‐0.76)	14.1
C_max_, ng/mL	13	329 (124)	310	13	519 (92)	515	0.60 (0.55‐0.66)	14.5

AUC_0‐12_, area under the plasma concentration–time curve from time 0 to 12 h; C_max_, maximum observed gefapixant concentration; CV, coefficient of variance; GMR, geometric mean ratio; LSM, least‐square mean; PPI, proton pump inhibitor; SD, standard deviation.

a For F02 25 mg every 12 h, once‐daily omeprazole 20 mg administered for 4 days 2 h before gefapixant dose; for F02 and F01 50 mg every 12 h, once‐daily omeprazole 20 mg administered for 2 days 2 h before gefapixant dose.

b The intrasubject CV percentage = 100√(e^[residual variance] − 1^).

In the fed state, the presence of omeprazole had less effect on gefapixant plasma concentrations with F02 compared with F01. Administration of omeprazole decreased AUC_0‐12_ and C_max_ by ≈30% to 40% compared with administration of F01 50 mg alone, whereas administration of omeprazole before either F02 25 or 50 mg did not significantly alter AUC_0‐12_ and C_max_ compared with F02 administration alone. The effect of omeprazole on F01 was slightly less than that seen in study 1.

The gefapixant formulations F02 and F01 50 mg every 12 hours demonstrated similar bioavailability in the fed state. Gefapixant F02 and F01 50 mg every 12 hours exhibited comparable geometric least‐square mean AUC_0‐12_ (3569 vs 3594 ng · h/mL) and C_max_ (511 vs 515 ng/mL), respectively. The F02/F01 GMR (90%CI) in the fed state for AUC_0‐12_ and C_max_ were 0.99 (0.90‐1.09) and 0.99 (0.90‐1.09), respectively.

### Formulation Bridging Gefapixant F02 to F04

#### Study 3

**Figure 4:**
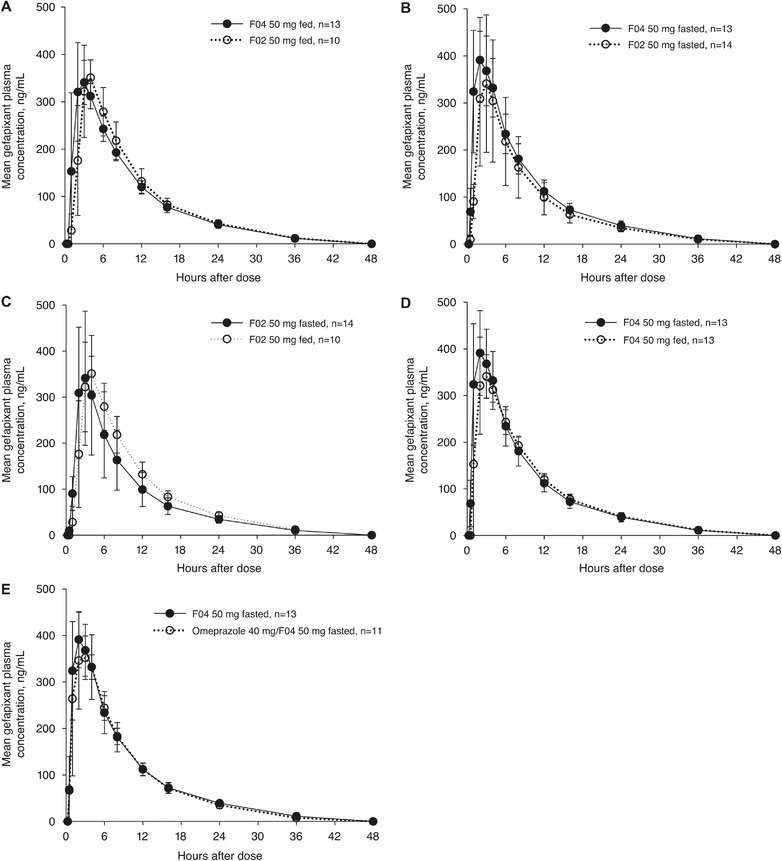
Mean (standard deviation) gefapixant plasma concentrations over time in healthy adult participants following administration of (A‐D) gefapixant F02 50 mg and F04 50 mg in fed or fasted states and (E) omeprazole 40 mg once daily for 5 days with gefapixant F04 50 mg administered 2 hours after omeprazole on day 5.

A total of 14 healthy participants were enrolled in the study; 4 failed to complete all 5 periods (1 participant was dismissed after period 1 because of a protocol violation, 2 participants were discontinued because of missed omeprazole dosing in period 4, and 1 participant did not return for period 5). Mean gefapixant plasma concentration vs time curves are presented in Figure 4; AUC, C_max_, t_max_, and t_1/2_ values are presented in Table 5. Consistent with F02 150‐mg dosing results from study 1 (Table 3), mean gefapixant AUC_0‐12_ and C_max_ after a single dose of F02 50 mg were higher in fed vs fasted conditions. Under fed conditions, a single dose of F04 50 mg resulted in similar gefapixant plasma AUC_0‐12_ and C_max_ as F02 50 mg. In fasted conditions, mean gefapixant plasma AUC_0‐∞_, AUC_0‐12_, and C_max_ were increased by 16%, 32%, and 32%, respectively, after administration of a single dose of F04 50 mg compared with a single dose of F02 50 mg.

**Table 5:** Summary of Statistical Comparisons of Gefapixant Pharmacokinetics for Formulations F02 and F04 After Administering Gefapixant With or Without Omeprazole to Healthy Adult Participants (Assessed in Fed and Fasted States)

Parameter	n	Arithmetic Mean (SD)	Geometric Mean (Test)	n	Arithmetic Mean (SD)	Geometric Mean (Reference)	GMR (90%CI)	Pseudo Within‐ Subject CV, %^a^
	**F04 50 mg (fasted)**			**F02 50 mg (fasted)**			**F04/F02**	
AUC_0‐∞_, ng · h/mL	13	3980 (661)	3950	12	3470 (976)	3410	1.16 (0.99‐1.35)	20.9
AUC_0‐12_, ng · h/mL	13	2740 (473)	2710	14	2280 (892)	2050	1.32 (1.01‐1.74)	39.9
C_max_, ng/mL	13	417 (95)	408	14	351 (152)	308	1.32 (1.00‐1.76)	42.4
t_1/2_, h	13	7.94 (1.00)	…	12	8.00 (1.75)	…	…	…
t_max_, h^b^	13	2.00 (1.00, 4.00)	…	14	3.00 (2.00, 3.00)	…	…	…

	**F04 50 mg (fed)**			**F02 50 mg (fed)**			**F04/F02**	
AUC_0‐∞_, ng · h/mL	13	3820 (434)	3810	10	3850 (455)	3890	0.98 (0.93‐1.04)	7.5
AUC_0‐12_, ng · h/mL	13	2540 (284)	2530	10	2500 (259)	2510	1.01 (0.95‐1.07)	7.6
C_max_, ng/mL	13	383 (68)	378	10	379 (52)	380	1.00 (0.91‐1.09)	12.9
t_1/2_, h	13	7.67 (0.71)	…	10	7.33 (0.55)	…	…	…
t_max_, h^b^	13	2.00 (1.00, 4.00)	…	10	3.00 (2.00, 4.00)	…	…	…

	**F04 50 mg (fed)**			**F04 50 mg (fasted)**			**Fed/Fasted**	
AUC_0‐∞_, ng · h/mL	13	3820 (434)	3810	13	3980 (661)	3950	0.96 (0.90‐1.04)	10.2
AUC_0‐12_, ng · h/mL	13	2540 (284)	2530	13	2740 (473)	2710	0.93 (0.86‐1.00)	10.8
C_max_, ng/mL	13	383 (68)	378	13	417 (95)	408	0.93 (0.83‐1.04)	16.5
t_1/2_, h	13	7.67 (0.71)	…	13	7.94 (1.00)	…	…	…
t_max_, h^b^	13	2.00 (1.00, 4.00)	…	13	2.00 (1.00, 4.00)	…	…	…

	**F02 50 mg (fed)**			**F02 50 mg (fasted)**			**Fed/Fasted**	
AUC_0‐∞_, ng · h/mL	10	3850 (455)	3890	12	3470 (976)	3410	1.14 (0.97‐1.34)	21.6
AUC_0‐12_, ng · h/mL	10	2500 (259)	2510	14	2280 (892)	2050	1.22 (0.92‐1.65)	41.3
C_max_, ng/mL	10	379 (52)	380	14	351 (152)	308	1.23 (0.92‐1.65)	42.8
t_1/2_, h	10	7.33 (0.55)	…	12	8.00 (1.75)	…	…	…
t_max_, h^b^	10	3.00 (2.00, 4.00)	…	14	3.00 (2.00, 3.00)	…	…	…

	**F04 50 mg + omeprazole 40 mg** **^c^** **(fasted)**			**F04 50 mg (fasted)**			**With PPI/Without PPI**	
AUC_0‐∞_, ng · h/mL	11	3800 (507)	3830	13	3980 (661)	3950	0.97 (0.90‐1.05)	10.6
AUC_0‐12_, ng · h/mL	11	2660 (401)	2640	13	2740 (473)	2710	0.97 (0.90‐1.05)	11.5
C_max_, ng/mL	11	377 (62)	373	13	417 (95)	408	0.91 (0.82‐1.02)	15.8
t_1/2_, h	11	7.38 (1.10)	…	13	7.94 (1.00)	…	…	…
t_max_, h^b^	11	3.00 (1.00, 4.00)	…	13	2.00 (1.00, 4.00)	…	…	…

AUC_0‐∞_, area under the plasma concentration–time curve from time 0 extrapolated to infinity; AUC_0‐12_, area under the plasma concentration–time curve from time 0 to 12 h; C_max_, maximum gefapixant plasma concentration; CV, coefficient of variance; GMR, geometric mean ratio; PPI, proton pump inhibitor; SD, standard deviation; t_1/2_, terminal half‐life; t_max_, time to C_max_.

a Pseudo within‐subject CV percentage = 100*√[(σC^2^ + σE^2^ − 2*σCE)/2], where σC^2^ and σE^2^ are the estimated variances on the log scale for the 2 treatment groups, and σCE is the corresponding estimated covariance, each obtained from the linear mixed‐effects model.

b Median (minimum, maximum) reported for t_max_.

c Omeprazole 40 mg once daily for 5 days with F04 50 mg on day 5.

In contrast to F02, there was no meaningful effect of food on mean AUC_0‐∞_, AUC_0‐12,_ or C_max_ after administration of a single dose of F04 50 mg (Table 5). Additionally, there was no meaningful effect of once‐daily omeprazole 40 mg administered for 5 days before a single dose of F04 50 mg on mean AUC_0‐∞_, AUC_0‐12,_ and C_max_ (Table 5).

### Formulation Bridging Gefapixant F04A to F04B

#### Study 4

A total of 40 healthy participants were enrolled and all completed the study. Mean gefapixant plasma concentration vs time curves are presented in Figure S1; AUC, C_max_, t_max_, and t_1/2_ values are presented in Table 6. The 90%CIs of the true GMRs (F04B 15 mg/F04A 15 mg and F04B 45 mg/F04A 45 mg) for all 3 PK parameters (gefapixant AUC_0‐∞_, AUC_0‐last_, and C_max_) were within the bounds of 0.80 and 1.25, thereby demonstrating bioequivalence between F04B 15 and 45 mg compared with the respective doses of F04A.

**Table 6:** Summary of Statistical Comparisons of Gefapixant Pharmacokinetics for Formulations F04A and F04B After Administering Gefapixant to Healthy Adult Participants (Assessed in the Fasted State)

Parameter	n	Arithmetic Mean (SD)	Geometric Mean	n	Arithmetic Mean (SD)	Geometric Mean	GMR (F04B/F04A) (90%CI)	Pseudo Within‐ Subject CV, %^a^
	**F04B 45 mg**			**F04A 45 mg**				
AUC_0‐∞_, ng · h/mL	20	3430 (797)	3350	19^b^	3570 (684)	3460	0.97 (0.93‐1.01)	7.0
AUC_0‐last_, ng · h/mL	20	3280 (781)	3190	19^b^	3410 (672)	3290	0.97 (0.93‐1.01)	7.0
C_max_, ng/mL	20	431 (127)	413	19^b^	463 (136)	435	0.95 (0.86‐1.04)	16.6
t_1/2_, h	20	6.73 (1.04)	…	19^b^	6.77 (1.07)	…	…	…
t_max_, h^c^	20	1.77 (1.00, 4.01)	…	19^b^	2.00 (1.00, 3.00)	…	…	…

	**F04B 15 mg**			**F04A 15 mg**				
AUC_0‐∞_, ng · h/mL	19^d^	1110 (186)	1100	20	1100 (220)	1080	1.01 (0.98‐1.06)	6.9
AUC_0‐last_, ng · h/mL	19^d^	996 (175)	984	20	988 (204)	968	1.02 (0.97‐1.06)	8.3
C_max_, ng/mL	19^d^	142 (34)	139	20	139 (32)	136	1.02 (0.96‐1.08)	10.5
t_1/2_, h	19^d^	5.85 (2.28)	…	20	5.22 (1.23)	…	…	…
t_max_, h^c^	19^d^	2.00 (1.07, 3.00)	…	20	1.51 (1.02, 3.00)	…	…	…

AUC_0‐∞_, area under the plasma concentration–time curve from time 0 extrapolated to infinity; AUC_0‐last_, area under the plasma concentration–time curve from time 0 to the time of the last quantifiable sample; C_max_, maximum gefapixant plasma concentration; CV, coefficient of variance; GMR, geometric mean ratio; SD, standard deviation; t_1/2_, terminal half‐life; t_max_, time to C_max_.

a Pseudo within‐subject CV percentage = 100*√[(σC^2^ + σE^2^ − 2*σCE)/2], where σC^2^ and σE^2^ are the estimated variances on the log scale for the 2 treatment groups, and σCE is the corresponding estimated covariance, each obtained from the linear mixed‐effects model.

b One participant did not return for period 2 dosing with F04A 45 mg.

c Median (minimum, maximum) reported for t_max_.

d One participant was excluded from the results for F04B 15 mg in this primary analysis because no samples could be collected for this participant at 1 and 1.5 h after dosing.

### Safety and Tolerability

The safety profile across all studies was generally consistent with previously published gefapixant data.^9^, ^17^ The most commonly reported AEs in studies 1 and 2 were taste related, and the most common AE in studies 3 and 4 was headache. There were no serious AEs reported in any of the studies, and all AEs were mild or moderate in severity.

## Discussion

Gefapixant is a P2X3‐receptor antagonist being developed for the treatment of RCC or UCC. Recently, gefapixant demonstrated significant reductions in 24‐hour cough frequency and improvement in cough‐related quality of life in 2 large, global, phase 3, randomized, double‐blind, placebo‐controlled studies.^11^ Through the development program of gefapixant, a series of formulations has been developed and clinically assessed.^9^, ^10^, ^11^ The 4 studies presented here describe a strategy that bridged gefapixant formulations from early‐ through late‐stage development to the final commercial formulation of gefapixant. These bridging studies established that the changes in the API form and optimization of the drug product composition successfully eliminated significant food and PPI effects seen with the early formulations. Bioavailability after F01 administration was significantly affected by food and intragastric pH, as AUC_0‐12_ and C_max_ increases of up to ≈2‐fold were observed in the fed state and reductions of ≈2‐ to 7‐fold were observed in the presence of omeprazole. In contrast, addition of an acidulant (citric acid) to the original F01 free base formulation resulted in a formulation (F02) with similar bioavailability to F01 but improved formulation performance due to reduced sensitivity to food and PPI effects (maximum changes in AUC_0‐12_ and C_max_ were <1.5‐fold). Thus, using an acidified citric acid–based formulation of gefapixant reduced the effect of food and PPIs on gefapixant bioavailability.

The formulation F04 contained a citrate salt–based API, rather than the gefapixant free base, to ensure a stable drug product for commercialization and to avoid the potential presence of different API phases (ie, free base and salt form) in F02. Gefapixant bioavailability after F04 administration was similar to F02 when administered in fed conditions (GMR 90%CIs were within 0.80 and 1.25) but demonstrated increased bioavailability compared with F02 in fasted conditions. Differences in bioavailability between F02 and F04 were driven by differential food effects, as overall bioavailability of gefapixant was increased when F02 was administered in fed vs fasted conditions, whereas there was no meaningful observed food effect on gefapixant bioavailability after F04 administration. The bioavailability of F04 was also not meaningfully affected by coadministration of omeprazole. After establishing a lack of food and gastric pH effects on the bioavailability of gefapixant F04, the formulation was adjusted for potency and improved for drug product processability.

The F04A formulation was developed with minor excipient differences from F04 that were not anticipated to affect drug absorption or dissolution, including changes to the lubricant levels and film coating composition. Given the insignificant nature of the formulation changes, multimedia pH dissolution was used to bridge F04 to F04A. The 2 formulations exhibited similar dissolution profiles in multimedia dissolution at pH levels of 1.2, 5.0, and 6.8 (≥85% dissolution in 15 minutes for both formulations at all pH levels; Figure S2). Tablet elegance issues with F04A necessitated changes in the grade of an excipient and removal of another excipient from the formulation, and the F04B formulation was subsequently developed as a final market formulation, which is devoid of the elegance issues identified with F04A. Because removal of an excipient constitutes a level 3 change per Scale‐up and Post‐Approval Changes guidance, study 4 was conducted to establish bioequivalence between the F04A formulation used in phase 3 studies and the final marketed F04B formulation in the fasted state; clinical bioequivalence between F04A and F04B was established at 15‐ and 45‐mg doses via comparability of gefapixant plasma pharmacokinetics.

In summary, the formulation development of gefapixant evolved from an original free base form, with bioavailability sensitivities to food and pH effects, to phase 3 and commercial formulations using the citrate salt, with bioavailability that is unaffected by the presence of food and PPI coadministration.

This study was supported by Merck Sharp & Dohme Corp., a subsidiary of Merck & Co., Inc., Kenilworth, New Jersey.

## Conflicts of Interest

P.G., A.H., J.C.N., A.S., and M.I. are employees of Merck Sharp & Dohme Corp., a subsidiary of Merck & Co., Inc., Kenilworth, New Jersey, and may hold stock or stock options in Merck & Co., Inc. A.P.F. is a former employee of Afferent Pharmaceuticals, Inc. (now acquired by Merck Sharp and Dohme Corp.) and may hold stock or stock options in Merck & Co., Inc. S.S. has nothing to disclose.

## Author Contributions

All authors provided final approval of the version to be published and agree to be accountable for all aspects of the work in ensuring that questions related to the accuracy or integrity of any part of the work are appropriately investigated and resolved.

## Supporting information



Supporting information

Supporting information
