# Supplementary material for: Clinical Formulation Bridging of Gefapixant, a P2X3‐Receptor Antagonist, for the Treatment of Chronic Cough
Source: Clin Pharmacol Drug Dev. 2022 May 5;11(9):1054–67. doi: 10.1002/cpdd.1105 (PMC9540877; doi:10.1002/cpdd.1105)
Supplement: Supplementary file 1 — Supporting information [file CPDD-11-1054-s001.pdf]

SUPPLEMENTAL TABLE

Supplemental Table. Participant Demographics[a]

Parameter	Study 1 (n=18)	Study 2 (n=14)	Study 3 (n=14)	Study 4; Part 1 (n=20)	Study 4; Part 2 (n=20)
Sex, n (%)					
Male	13 (72)	8 (57)	9 (64)	4 (20)	5 (25)
Female	5 (28)	6 (43)	5 (36)	16 (80)	15 (75)
Age, median (range), y	32 (20, 51)	29 (19, 52)	33 (21, 58)	34 (19, 48)[b]	36 (22, 50)[b]
BMI, mean (SD), kg/m^2	27.4 (3.9)	24.5 (3.4)	27.3 (3.0)	26.4 (3.1)	27.4 (2.7)
Race, n (%)					
American Indian/Alaskan Native	2 (11)	0	0	0	0
Asian	0	0	0	0	1 (5)
Black or African American	6 (33)	0	11 (79)	2 (10)	1 (5)
Native Hawaiian/Pacific Islander	1 (5)	0	0	0	0
White	9 (50)	13 (93)	3 (21)	17 (85)	18 (90)
Other	0	1 (7)	0	1 (5)	0
Ethnicity, n (%)					
Hispanic or Latino	5 (28)	5 (36)	0	16 (80)	13 (65)
Not Hispanic or Latino	13 (72)	9 (64)	14 (100)	4 (20)	7 (35)

BMI, body mass index. [a]Includes all participants included in safety analyses. [b]Mean (range) age.
