# Supplementary material for: Clinical Formulation Bridging of Gefapixant, a P2X3‐Receptor Antagonist, for the Treatment of Chronic Cough
Source: Clin Pharmacol Drug Dev. 2022 May 5;11(9):1054–67. doi: 10.1002/cpdd.1105 (PMC9540877; doi:10.1002/cpdd.1105)
Supplement: Supplementary file 2 — Supporting information [file CPDD-11-1054-s002.pdf]

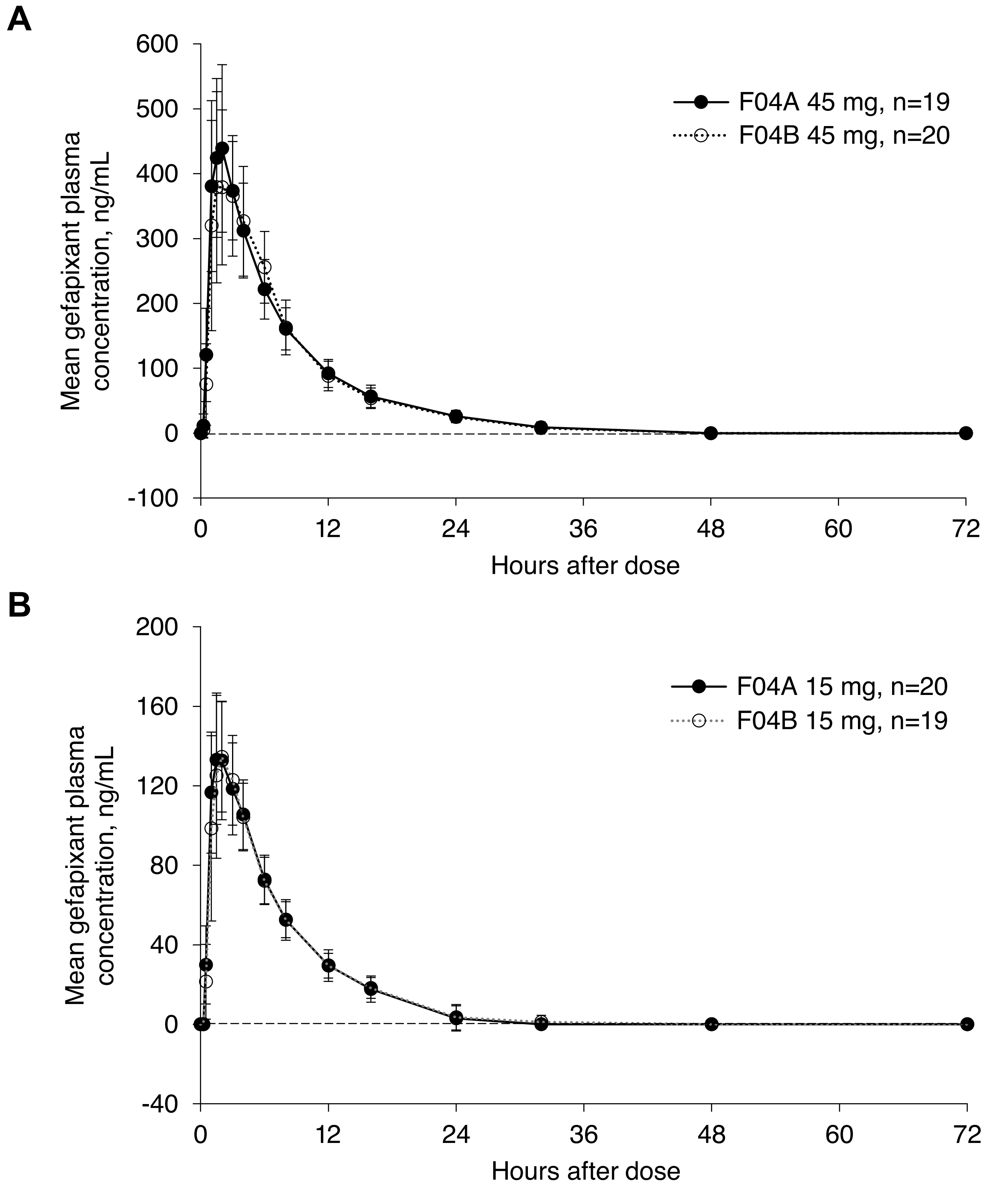

Supplemental Figure 1. Mean (SD) gefapixant plasma concentrations over time in healthy adult participants following administration of a single dose of gefapixant **(A)** F04A and F04B 45 mg or **(B)** F04A and F04B 15 mg in the fasted state.

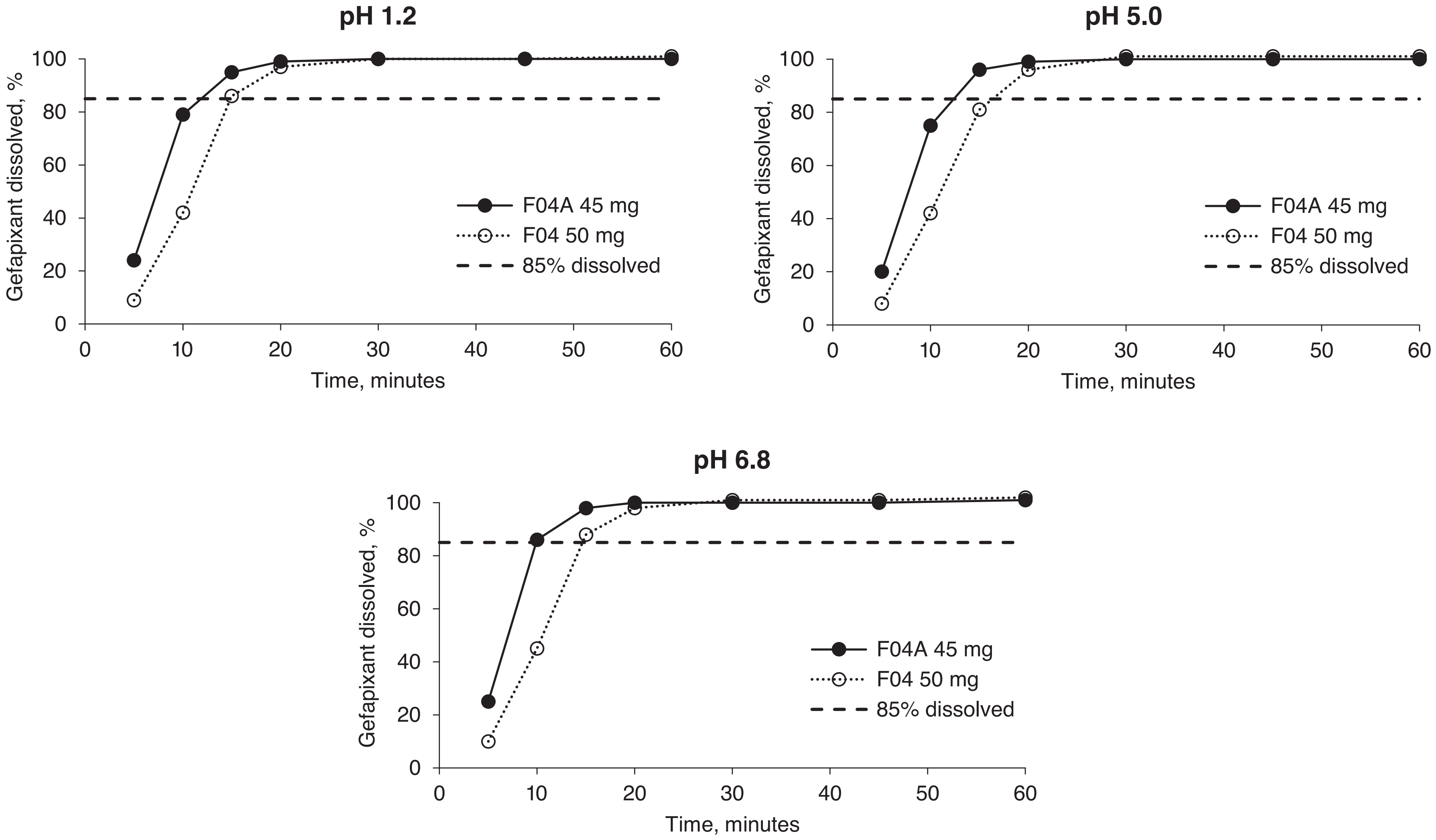

Supplemental Figure 2. Multimedia dissolution profiles of F04A 45 mg and F04 50 mg at pH levels 1.2, 5.0, and 6.8. Data represent the mean percentage dissolved of n=12 coated tablets for each group.
